# Effectiveness of interventions on early neurodevelopment of preterm infants: a systematic review and meta-analysis

**DOI:** 10.1186/s12887-021-02559-6

**Published:** 2021-04-29

**Authors:** Marilyn Aita, Gwenaëlle De Clifford Faugère, Andréane Lavallée, Nancy Feeley, Robyn Stremler, Émilie Rioux, Marie-Hélène Proulx

**Affiliations:** 1 Faculty of Nursing, Université de Montréal, P.O. Box 6128, Succursale centre-ville, Montréal, QC H3C 3J7 Canada; 2 CHU Sainte-Justine Research Centre, 3175, chemin de la Côte-Sainte-Catherine, Montréal, QC H3T 1C5 Canada; 3 Quebec Network on Nursing Intervention Research, PO Box 6128, Centre-ville, Montréal, QC H3C 3J7 Canada; 4 Ingram School of Nursing, McGill University, 680 Rue Sherbrooke Ouest #1800, Montréal, QC H3A 2M7 Canada; 5 Centre for Nursing Research and Lady Davis Institute, Jewish General Hospital, 3755 Chemin de la Côte-Sainte-Catherine, Montréal, QC H3T 1E2 Canada; 6 Lawrence S. Bloomberg Faculty of Nursing, University of Toronto, 155 College St, Toronto, ON M5T 1P8 Canada; 7 Hospital for Sick Children (SickKids), 555 University Ave, Toronto, ON M5G 1X8 Canada; 8 Jewish General Hospital, 3755 Chemin de la Côte-Sainte-Catherine, Montréal, QC H3T 1E2 Canada

**Keywords:** Neurodevelopment, Preterm infants, Systematic review, Interventions, NICU, Meta-analysis

## Abstract

**Background:**

As preterm infants’ neurodevelopment is shaped by NICU-related factors during their hospitalization, it is essential to evaluate which interventions are more beneficial for their neurodevelopment at this specific time. The objective of this systematic review and meta-analysis was to evaluate the effectiveness of interventions initiated during NICU hospitalization on preterm infants’ early neurodevelopment during their hospitalization and up to two weeks corrected age (CA).

**Methods:**

This systematic review referred to the Preferred Reporting Items for Systematic Reviews and Meta-Analyses [PRISMA] guidelines and was registered in PROSPERO (CRD42017047072). We searched CINAHL, MEDLINE, PubMed, EMBASE (OVID), Cochrane Systematic Reviews, CENTRAL, and Web of Science from 2002 to February 2020 and included randomized controlled/clinical trials conducted with preterm infants born between 24 and 36^6/7^ weeks of gestation. All types of interventions instigated during NICU hospitalization were included. Two independent reviewers performed the study selection, data extraction, assessment of risks of bias and quality of evidence.

**Results:**

Findings of 12 studies involving 901 preterm infants were synthesized. We combined three studies in a meta-analysis showing that compared to standard care, the NIDCAP intervention is effective in improving preterm infants’ neurobehavioral and neurological development at two weeks CA. We also combined two other studies in a meta-analysis indicating that parental participation did not significantly improve preterm infants’ neurobehavioral development during NICU hospitalization. For all other interventions (i.e., developmental care, sensory stimulation, music and physical therapy), the synthesis of results shows that compared to standard care or other types of comparators, the effectiveness was either controversial or partially effective.

**Conclusions:**

The overall quality of evidence was rated low to very low. Future studies are needed to identify interventions that are the most effective in promoting preterm infants’ early neurodevelopment during NICU hospitalization or close to term age. Interventions should be appropriately designed to allow comparison with previous studies and a combination of different instruments could provide a more global assessment of preterm infants’ neurodevelopment and thus allow for comparisons across studies.

**Trial registration:**

Prospero CRD42017047072.

**Supplementary Information:**

The online version contains supplementary material available at 10.1186/s12887-021-02559-6.

## Background

### Rationale

Over the last decades, the increasing survival rates of preterm infants admitted in the Neonatal Intensive Care Unit [NICU] have emphasized the importance of considering their neurodevelopmental outcomes [1]. Preterm infants are more likely to have significant short- and long-term neurodevelopmental impairments, since their brains go through a critical period of development and maturation between 24 and 40 weeks of gestation occurring mainly during their NICU hospitalization [2, 3]. Significant neurodevelopmental disabilities associated with neurosensory, motor, behavioral and cognitive outcomes may then arise shortly after preterm infants’ birth and last beyond school age and adolescence [4] given that neurocognitive impairments in infants born preterm are still reported in adulthood [5].

Many factors in the NICU environment may be congruent or incongruent with the preterm infants’ developing brain and consequently influence their neurodevelopmental outcomes. Environmental stimulation (i.e., light, noise) [2, 6–8], social interactions with parents [7, 9] and caregiving experience [2] are among influential environmental NICU factors. Interventions targeting these elements during NICU hospitalization should therefore foster preterm infants’ early neurodevelopment.

Different interventions initiated during NICU hospitalization targeting some of these influential NICU factors with the objective of improving preterm infants’ neurodevelopment have been examined in previous systematic reviews. These interventions consist of developmental care a wide-ranging group of interventions to minimize preterm infants’ NICU environmental stress [10–12], including Neonatal Individualized Developmental Care Assessment Program [NIDCAP], which is an individualized approach based on observing preterm infants’ behaviors to guide caregiving activities provided by professionals and infants’ family [13], noise reduction management, [14] in addition to interventions with parental involvement (i.e. NIDCAP, kangaroo care and developmental care) [15] and skin-to-skin contact [16]. The effectiveness of these interventions has been evaluated on the long-term neurodevelopment of preterm infants at 12 months or 24 months of age, providing important evidence. Yet, measuring the effects of interventions implemented during hospitalization only at one year of age and older raises concerns about confounding factors that may have occurred between NICU discharge and the timing of the assessments and that could have influenced the infants’ long-term neurodevelopment along with interventions.

Neurodevelopmental assessments of preterm infants during NICU hospitalization are currently performed to predict long-term neurodevelopmental outcomes and plan early interventions [17–19]. A systematic review confirmed that neurobehavioral assessments of infants’ general movements (GMs) referring to the most common and complex spontaneous movements patterns [20] and infants’ posture and movements with the Test of Infant motor Performance (TIMP) [21] done before term corrected age [CA] are the most significant predictors of long-term neurodevelopment [18]. Nonetheless, to our knowledge, no systematic review has evaluated the effectiveness of interventions on the preterm infants’ short-term neurodevelopment, that is, during NICU hospitalization or close to term CA. As preterm infants’ neurodevelopment is shaped by NICU-related factors as soon as they are hospitalized, it is imperative to explore which interventions are most beneficial for their neurodevelopment at this specific time encompassing a decisive period for their brain development and maturation.

### Objective

The primary objective of this systematic review and meta-analysis was to evaluate the effectiveness of interventions initiated during NICU hospitalization and delivered by healthcare professionals and/or parents on preterm infants’ early neurodevelopment during their NICU hospitalization and by two weeks CA.

## Methods

### Protocol and registration

This systematic review protocol was published [22] and registered in the International Prospective Register of Systematic Reviews [PROSPERO] (CRD42017047072). The systematic review follows the Preferred Reporting Items for Systematic Reviews and Meta-Analyses [PRISMA] guidelines [23].

The published protocol [22] was modified for the search strategy, as we did not manually search journals. Journals focusing on neonatology, neonates and/or neurodevelopment were already indexed, thus articles published in these journals were captured by our searches in various databases. It was initially planned to only include studies measuring preterm infants’ neurodevelopment during NICU hospitalization, but we also considered studies assessing this outcome at infants’ two weeks CA since it was sufficiently close to NICU hospitalization. We included as well studies where preterm infants in study samples had an intraventricular hemorrhage [IVH] greater than grade II [24–26] and various brain injuries (i.e., IVH, cerebellar hemorrhage and periventricular leukomalacia) [27], which differed from the original protocol. These four studies were included as their samples were representative of the neonatal population and the infants’ baseline characteristics and physiologic stability were similar to those allocated to the experimental and control groups, suggesting that they were not significantly different because of IVH or brain injuries. We also included interventions with different components when they were specifically described as being provided together as a complete intervention.

### Eligibility criteria of the selected studies

We included randomized controlled/clinical trials [RCTs] and one pilot RCT conducted with preterm infants born between 24 and 36^6/7^ weeks of gestation. Interventions were instigated during NICU hospitalization and were delivered by healthcare professionals or parents, or both and all types of interventions were included. The studies included were written in English or French and were published in the past 18 years, from 2002 to February 2020. All types of comparator groups, such as non-exposed control group or a group exposed to different interventions, were included in this systematic review. Studies reporting preterm infants’ neurodevelopment as an outcome evaluated with a standardized instrument, scale or test were also considered.

### Search strategy and information sources

An expert librarian was consulted to conduct the search in the following electronic databases from 2002 to February 2020: CINAHL, MEDLINE, Pubmed, EMBASE [OVID], Cochrane Database of Systematic Reviews, Cochrane Central Register of Controlled Trials [CENTRAL] and Web of Science (see Additional File 1, Table S1 for an example of a search strategy in MEDLINE). The Scopus database was also reviewed to search for trials in conference proceedings and ProQuest for theses and dissertations. We also looked for ongoing trials at clinicaltrials.gov, clinicaltrialsregister.eu, the World Health Organization International Clinical Trials Registry Platform (http://www.who.int/ictrp/en/), the International Standard Randomized.

Controlled Trial Number (ISRCTN) registry and the Australian New Zealand Clinical Trials Registry to identify trials that were underway. We also searched three grey literature websites: http://www.opengrey.eu/, http://opengrey.org/ and www.greylit.org. We manually checked the references of included studies.

### Study selection

All references for the studies selected for this review were managed in EndNote© X9. After removing duplicates, the screening was conducted by separate reviewers (MA, GDF, AL). The reviewers screened the remaining studies for eligibility by reviewing study titles and abstracts, then the full-text reports (GDF, ER, MHP) to evaluate their appropriateness to be included in the systematic review. For these steps, agreement was reached between two reviewers and disagreement was resolved by consensus with a third reviewer (MA).

### Data extraction

Data extraction was performed independently by two authors (ER, MHP) using a data extraction form and pilot tested with two studies [28]. The extracted data were compared for all included studies, and disagreement was solved with a third reviewer (GDF). The extracted information for each study was described in the protocol [22]. All the extracted data were recorded in Review Manager (RevMan) [computer program] (version 5.1 Copenhagen: The Nordic Cochrane Centre, The Cochrane Collaboration, 2014). The data were double-checked by two reviewers (GDF, AL) before conducting the analysis, to avoid errors.

### Data items

The primary outcome was preterm infants’ neurodevelopment assessed by a standardized instrument or scale during NICU hospitalization or at term-corrected age. For this systematic review, we considered neurobehavioral, neuromotor, neuromuscular and neurological development evaluation measured by the following instruments: preterm infants’ neurobehavioral development (Assessment of Preterm Infant Behavior – APIB; NICU Neonatal Neurobehavioral Scale – NNNS; Neonatal Neurobehavioral Examination – NNE), neuromotor development (Infant Neurological International Battery – INFANIB; Test of Infant Motor Performance – TIMP), neuromuscular development (New Ballard Score and Dubowitz examination) and neurological development (Prechtl Neurological Examination of the Full-term Newborn). Two of these instruments included subscales; six in the APIB: autonomic, motor, state, interaction-attentional, and self-regulation systems, in addition to the assessment of the degree of examiner facilitation necessary to support the infant’s reorganization when disorganized, and 13 in the NNNS: orientation, habituation, hypertonicity, hypotonicity, excitability, arousal, lethargy, nonoptimal reflexes, asymmetric reflexes, stress, self-regulation, quality of movement and handling. We considered measurements performed before NICU discharge and no later than two weeks after term CA. For measurements performed before term age, we considered the closest measure to discharge.

### Assessment of risk of bias of individual studies

The Cochrane risk of bias [28] assessment tool was used to determine the studies’ risk of bias, classified as low, high, or unclear risk of bias. The Cochrane tool considers sequence generation, allocation concealment, blinding, incomplete outcome data and selective outcome reporting. The risk of bias was assessed independently by three reviewers (GDF, ER, MHP). In the case of disagreement, consensus was reached by consulting two other reviewers (MA, AL).

### Summary measures

Statistical analyses were conducted with the Review Manager (RevMan) 5.1 software, using a random-effect model with a 95% confidence interval [CI]. Different instruments were used to measure neurodevelopment so to maintain homogenous data, we chose to treat each instrument separately. For continuous outcomes, mean differences [MD] were calculated from extracted means and standard deviations. For dichotomous outcomes (frequency and percentage), relative risk [RR] was reported.

### Synthesis of results

If two or more studies reported the same outcome with the same instrument, a meta-analysis was conducted with RevMan 5.1 using a random-effect model and inverse variance, as suggested for studies with heterogeneity [PRISMA], with a 95% CI. Heterogeneity was evaluated using the Chi-squared test with a significance level of 0.1 and the I^2^ using the classification suggested by PRISMA-P: 0 to 40%, not important heterogeneity; 30 to 60%, moderate heterogeneity; 50 to 90%, substantial heterogeneity; and 75,100%, considerable heterogeneity [29]. The unit of analysis in our systematic review consisted of preterm infants receiving an intervention or a control comparator during NICU hospitalization.

### Missing data

One author was contacted to obtain data [30]. As the author was not able to provide the data to allow for imputation, the study was excluded from the meta-analysis.

### Quality of evidence

The quality of evidence was assessed for each outcome using the Grading of Recommendations Assessment, Development and Evaluation (GRADE) guidelines [31] by three authors (MA, GDF, AL). The GRADE system rates the quality of evidence as high, moderate, low or very low in five areas: risk of bias, inconsistency, indirectness, imprecision and publication bias. We downgraded each area by one or two points based on judgment criteria. For risk of bias, we did not downgrade if most risk of bias judgments were rated as “low” and downgraded by one point if the majority were rated “unclear” or “high.” For inconsistency, we did not downgrade if heterogeneity was considered as not important (< 40%), and we downgraded by one point if there was moderate or substantial heterogeneity among the studies (40 to 75%). For indirectness of evidence, we did not downgrade for any outcome. For imprecision, we downgraded by one point if the total number of participants was lower than 400 for the assessed outcome and downgraded by two points if the number of participants was lower than 150. The summary of findings table was generated using the GRADE profiler Guideline Development Tool software and the GRADE criteria (2015, McMaster University and Evidence Prime Inc.).

## Results

### Study selection

**Fig. 1:**
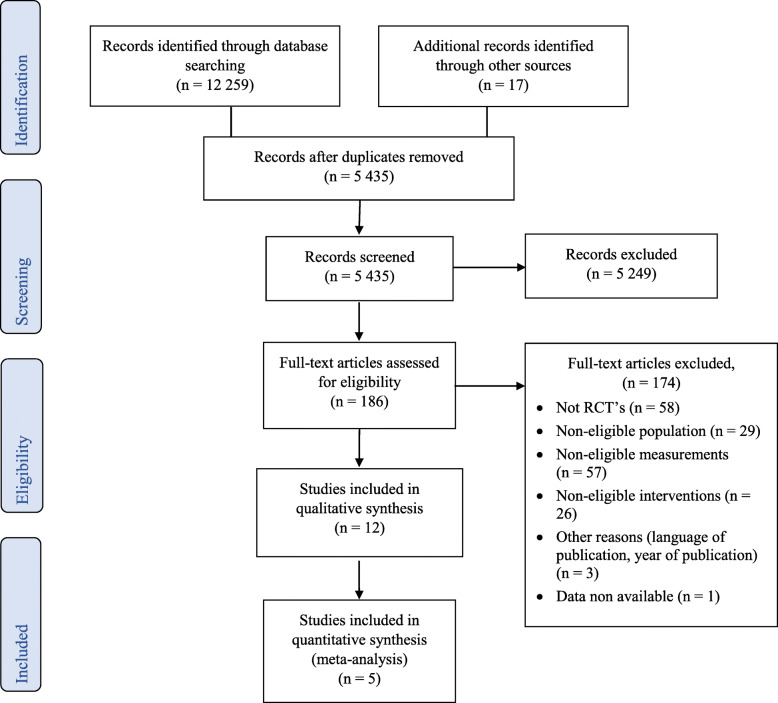
PRISMA Flow diagram

The study selection process is illustrated by the PRISMA Study Flow Diagram (see Fig. 1). Of the 12,259 screened studies, 186 were assessed for eligibility in the final selection. A total of 174 full-text articles were excluded for different reasons: a) 58 articles were not randomized controlled trials; b) 29 articles included exclusively preterm infants with brain abnormalities; c) 57 articles did not measure neurodevelopment or did not measure it with a standardized instrument; d) 26 articles were not eligible interventions; e) three articles were excluded for other reasons (language of publications other than French and English or year of publication before 2002); and f) data was not available for one article. Finally, 12 studies met the inclusion criteria and were included in this systematic review [24–27, 32–39]. Five studies were included in meta-analysis [24, 26, 32, 34, 38] for infants’ neurodevelopment. All the other studies were not suitable for meta-analysis because the nature of the intervention or the instrument used to measure neurodevelopment was different, precluding data pooling [25, 27, 33, 35–37, 39].

### Characteristics of included studies

The main characteristics of the included studies are summarized in Table 1. The 12 studies published between 2002 and February 2020 included 933 preterm infants. Five studies were conducted in the United States [24, 27, 32, 34, 36], one in the Netherlands [25], two in India [33, 35], one in Thailand [26], one in Taiwan [38], and two in Iran [37, 39]. Eleven studies were RCTs [24–27, 32–35, 37–39] and one was a pilot RCT with published results [36].

**Table 1:** Characteristics of included studies in systematic review

Author, year Country	Sample and participants (size, WGA and/or birthweight)	Study Design	Intervention Description	Intervention (who delivered, duration, dose, frequency)	Comparator group	Outcome measurement	Timing of assessment	Main Findings
Als, 2003 [24] USA	*N* = 92 < 28 WGA < 1250 g BW	**RCT** 2 parallel groups from 3 different centers.	**Developmental Care - NIDCAP** emphasizes the behavioral individuality of each infant. It seeks to diminish the infant’s experiences of stress and to enhance the infant’s strengths.	Delivered daily (7 days per wk) by two professionals (a NICU nurse and a developmental professional).	**Standard care** - some degree of shielding of incubators, sound containment, use of breast milk, and referral to occupational and physical therapy and to community early intervention. Kangaroo care (skin-to-skin holding) was used in one study center.	Neurobehavioral: **Assessment of Preterm Infant’s Behavior (APIB)**. **Measurements:** Autonomic (respiration, digestion, color), motor (tone, movement, postures), state organization (range, robustness, transition patterns), attention and self-regulation.	At 2 weeks’ CA Infant’s behaviors were recorded every 2 min for approximately 1 h during a medical, nursing, or parent caregiving activity.	Infants of the experimental group had enhanced neuro-behavioral development (for all APIB system scores) compared to infants of the comparator group.
Als, 2004 [32] USA	*N* = 30 28 to 33 WGA BW between 5 and 95%	**RCT** 2 parallel groups.	**Developmental care - NIDCAP** Developmental care emphasizes the behavioral individuality of each infant. Each infant is seen as an active participant in all care.	Initiated within 72 h of admission and continued to the age of 2 weeks CA. Delivered daily (7 days/wk) by two certified NIDCAP professionals, psychologist, and infant developmentalist.	**Standard care** - shielding incubators, early use of dressing in T-shirts, and side and foot rolls; liberal provision of pacifiers; and inconsistent nurse-dependent encouragement of skin-to-skin holding and breastfeeding.	Neurobehavioral: **APIB**. Neurological: **Precthl Neurological Examination of the Full-term Newborn**.	At 2 weeks’ CA.	Infants of the experimental group had enhanced neurobehavioral and neurological development (for all APIB system scores and Precthl variables) compared to infants of the comparator group.
Kanagasabai, 2013 [33] India	*N* = 50 28 to 36 WGA 1000-2000 g BW	**RCT** 2 parallel groups.	**Sensory Stmulation Multisensory - ATVV** stimulation program: **A**uditory - Soft lullaby between (30–40 dB) for 3 min using a miniature speaker and an mp3 player; **T**actile - Gentle stroking massage for 3 min in a sequence of chest, upper limbs and lower limbs in supine position; **V**isual - Black and white visual stimulation card hung at a distance of 8–10 in. from the neonate for 3 min; **V**estibular - Gentle horizontal and vertical rocking for 3 min.	Initiated when infants reached 33 weeks of GA and within 48 h of birth for infants born at 33–36 wk. Delivered five days per wk. × 12 min daily and until discharge from the hospital.	Usual care: Kangaroo mother care and exclusive breastfeeding.	Neuromotor development: **Infant Neurological International Battery (INFANIB)** **Measurements:** Spasticity, head and trunk, vestibular function, legs, French angles (scarf sign, heel to ear, popliteal angle, abductor’s angle).	At term age (between 38 to 40 weeks GA).	Infants in the experimental group had significantly improved neuromotor development compared to infants of the comparator group.
Madlinger-Lewis, 2014 [27] USA	N = 92 ≤ 32 WGA	**RCT** 2 parallel groups.	**Developmental Care - Alternative positioning:** Structured blanket (Dandle Roo) made of stretchable cotton with adjustable straps for the upper extremities, a pouch for the lower extremities, and a head boundary designed to hold the legs in a weight-bearing, flexed position, while allowing for movement with recoil back to flexion.	Delivered continuously by nurses and parents whenever in bed and not being held or fed until discharge.	Traditional positioning consisted of any positioning devices or adaptations without the use Dandle Roo (swaddling, use of blankets and cloth rolls).	Neurobehavioral: **NICU Network Neurobehavioral Scale (NNNS)**. **Measurements:** habituation (not evaluated), orientation, tolerance of handling, quality of movement, self regulation, non-optimal reflexes, stress signs, arousal, hypertonia, hypotonia, asymmetry, excitability, and lethargy.	Between 35 to 40 weeks PMA.	Infants in the experimental group showed significantly less asymmetry of reflex and motor responses than those in the comparator group. No significant effect was found between groups for the other NNNS subscales.
Maguire, 2008 [25] Netherlands	*N* = 192 <32 WGA	**RCT** 2 parallel groups.	**Developmental care – Positioning and incubators covers** reduction of light and sound through use of incubator covers and using positioning aids to optimize physiologic stability and motor development and promote nesting.	Delivered continuously within 48 h of life until discharge.	Usual care consisted of no covers or nesting.	Neurological: **Precthl Neurological Assessment of the Full-Term Newborn**.	At 40 weeks PMA.	No significant effect between the groups was found for infants’ neurological development.
McAnulty, 2009 [34] USA	*N* = 107 < 29 weeks WGA < 1250 g BW	**RCT** 2 parallel groups.	**Developmental Care – NIDCAP**	Initiated upon NICU admission and continued to the age of 2 weeks CA. Delivered daily (7 days per wk) by two certified NIDCAP professionals, an nurse and a psychologist.	Control group received standard NICU care.	Neurobehavioral: **APIB**. Neurological: **Precthl Neurological Assessment of the Full-Term Newborn**.	Weekly observations throughout hospital stay to 2 weeks CA.	Infants of the experimental group had enhanced neurobehavioral development (for 5 APIB scores) and a trend towards better performance on the neurological assessement (Precthl) compared to infants of the comparator group.
Nakwa, 2017 [35] India	*N* = 36 33 WGA (mean)	**RCT** 2 parallel groups.	**Music**: Lullaby (30 to 40 dB during 30 min. Preterm infants received the developmental program as a standard of care.	Delivered three times a week × 30 min for a total of 3 weeks.	**Developmental program** (as a standard of care) including a tactile, visual, auditory and vistubural stimulation.	Neuromotor development: **Test of Infant Motor Performance (TIMP)** (posture and movements). Neurological devlopment: **INFANIB**.	First and last day of intervention during hospitalization.	Infants in the experimental group had significantly improved neuromotor and neurological development compared to infants of the comparator group.
Namprom, 2018 [26] Thailand	N = 50 28 to 32 WGA < 2500 g BW	**RCT** 2 parallel groups.	**Parental participation program (maternal):** Three key components: 1- Psychosocial support for mothers to participate in their infant’s care 2-Parent education: teaching the content of developmental care practices for preterm infants 3-Therapeutic developmental interventions pertaining to performance of care practices.	Delivered by a nurse and mothers. Activities were four 1-h teaching sessions and 4 1-h practice sessions over 4 weeks.	**Standard care** (no description mentioned).	Neurobehavioral development: **Neonatal Neurobehavioral Examination (NNE)**. **Measurements:** Tone and motor patterns, primitive reflexes, and behavioral responses.	At day 14 and 28 after infants’ birth.	Infants in the experimental group had significantly better neuro-behavioral development (tone and motor pattern, behavioral responses and total score) compared to infants of the comparator group.
Smith, 2014 [36] USA	*N* = 20 < 30 WGA < 1000 g BW	**Pilot RCT** 2 parallel groups.	**Sensory Stimulation – Tactile (Relaxation (M technique)** Structured touch of 8 distinctive patterns, stroking preterm infant’s back on each side of the spine (with the pads of the third and fourth fingers of both hands).	Initiated when the infant reached 30 weeks of GA. Delivered by a nurse or a another research team member. A 7 min of the M technique, 6 times per week for a total of 5 weeks.	**Standard care** in the two groups (family-centered care and neurodevelopmental strategies including parental presence, skin-to-skin contact, adequate positioning, environment supporting sleep).	Neurobehavioral development: **NNNS** Habituation (not evaluated in study), orientation, tolerance of handling, quality of movement, self-regulation, nonoptimal reflexes, stress signs, arousal, hypertonia, hypotonia, asymmetry, excitability, and lethargy.	At 35 weeks PMA at the end of the 5 weeks of intervention.	No significant difference between groups was found for any of the NNNS subscales.
Valizadeh, 2017 [37] Iran	N = 76 25 to 30 WGA 1000 g to 2000 g BW	**RCT,** 4 parallel groups Containement group, combination group, hydrotherapy group, physical activity group.	**Three interventions** **1. Hydrotherapy group** Preterm infant placed in water. Head, neck and pelvis supported **(**Sweeney and Vignochi method). **2. Physical activity group**: Extension and flexion: wrist, elbow, shoulder, ankle, knee and hip joints (Moyer-Mileur protocol). **3. Combination group** Hydrotherapy and physical activity.	Initiated at 32 weeks GA. Delivered by a nurse. For each 3 interventions delivered 10 min daily (30 min or 1 h before feeding) for 14 days.	Containment group: Preterm infants placed in a fetal position (lateral) with one hand on the preterm infant’s head (at the top) and one hand over the trunk and hip area,	Neuromotor development: **TIMP**. Neuromuscular development: **New Ballard Score and 2 items from Dubowitz examination**. **Measurements:** **New Ballard Score:** Posture, Arm Recoil, Popliteal Angle, Scarf Sign, Heel to Ear, Square window. **Items from Dubowitz examination:** Leg recoil and ankle dorsiflexion.	At 34 weeks PMA (post-intervention).	No significant effect between groups was found for’ neuromotor and neuromuscu-lar development. Infants of the physical therapy and hydrotherapy groups had significantly better leg recoil from Dubowitz examination compared to infants of the comparator group.
Yu, [38] 2019 Taiwan	N = 76 32 to 36 WGA < 1500 g BW	**RCT,** 2 parallel groups.	**Parental Participation Program**: interventions on parental participation, feeding, massage, activities, parental support, home transition.	Delivered by physical therapists, parents and nurses. During the NICU stay 5 sessions of interventions of one hour.	Standard care: 5 interventions in the NICU (according to the synactive theory of development) and 7 phone calls after discharge.	Neurobehavioral development: **NNE** (Chinese version).	Around 40 weeks PMA (between 38 to 44 weeks).	Infants in the experimental group had significantly better neuro-behavioral development (tone and motor patterns and total score) than infants of the comparator group.
Zeraati, [39] 2018 Iran	*N* = 80 32 to 36 WGA	**RCT,** 2 parallel groups.	**Sensory Stimulation - Multisensory** Auditory stimulation (lullaby, 30 to 40 dB for 3 min), tactile stimulation (3 min massage), visual stimulation (3 min black an white card), vestibular stimulation (gentle rockin for 3 min).	Initiated 48 h after birth. Delivered 12 min/session and 5 sessions per week until NICU discharge.	Standard care.	Neuromuscular development: **New Ballard Score.**	At discharge.	Infants of the experimental group had significantly better neuromuscu-lar development after the intervention compared to infants of the comparator group.

More than half the studies (*n* = 7) included preterm infants born younger than or at 32 weeks of gestational age [24–27, 34, 36, 37]. Five studies included infants born at a gestational age higher than 32 weeks [32, 33, 35, 38, 39].

The 12 studies include a variety of interventions, including NIDCAP [24, 32, 34], positioning and incubators covers [25], alternative positioning [27], sensory stimulation interventions considering tactile stimulation [36] and multisensory stimulation [33, 35], parental participation programs [26, 38], music [39] and physical activity and/or hydrotherapy [37].

Four studies did not specify who performed the intervention [25, 33, 35, 39]. For three studies, the intervention was delivered by two certified NIDCAP professionals [24, 32, 34]. The other interventions were either performed by a nurse [37], mothers with guidance from nurses [26], nurses and/or parents when they were at the bedside [27], the principal investigator (a nurse) or a trained research team member [36], or physical therapists, parents and nurses [38]. The majority of the studies (*n* = 8) comprehensively described their control group (i.e., standard care or comparator group) [24, 25, 27, 32, 33, 35–38], and three that described their control group as standard care did not provide specifics [26, 34, 39].

The shortest intervention duration was 14 consecutive days [37], whereas the longest was five weeks, with the intervention performed six days per week [36]. In some studies, the intervention was performed at a distinctive dose and frequency during NICU hospitalization [26, 33, 35–39] (see Table 1 for characteristics of studies). Four interventions were carried out for short periods of time ranging from five to 15 min [33, 36, 37, 39], 30 min [35] or one hour [38], whereas the others were almost always regularly integrated into care, with no details about frequency or dose [24, 25, 27, 32, 34].

The studies measured neurodevelopment using different scales, such as the APIB [24, 32, 34], the Prechtl Neurological Examination of the Full-term Newborn [25, 32, 34], the INFANIB [33, 35], the NNNS [27, 36] the TIMP [35, 37], the NNE [26, 38], the New Ballard Score [37, 39] and items from the Dubowitz examination [37]. Four studies used more than one scale [32, 34, 35, 37]. Eight studies included preterm infants whose neurodevelopment was measured during NICU hospitalization or at discharge [25–27, 33–39] and three studies measured the primary outcome at two weeks CA [24, 32, 34]. Three studies did their measurements at the end of the intervention [35–37], while one study performed their last measurement 28 days after the infants’ birth rather than at the end [26].

### Risk of bias of included studies

**Fig. 2:**
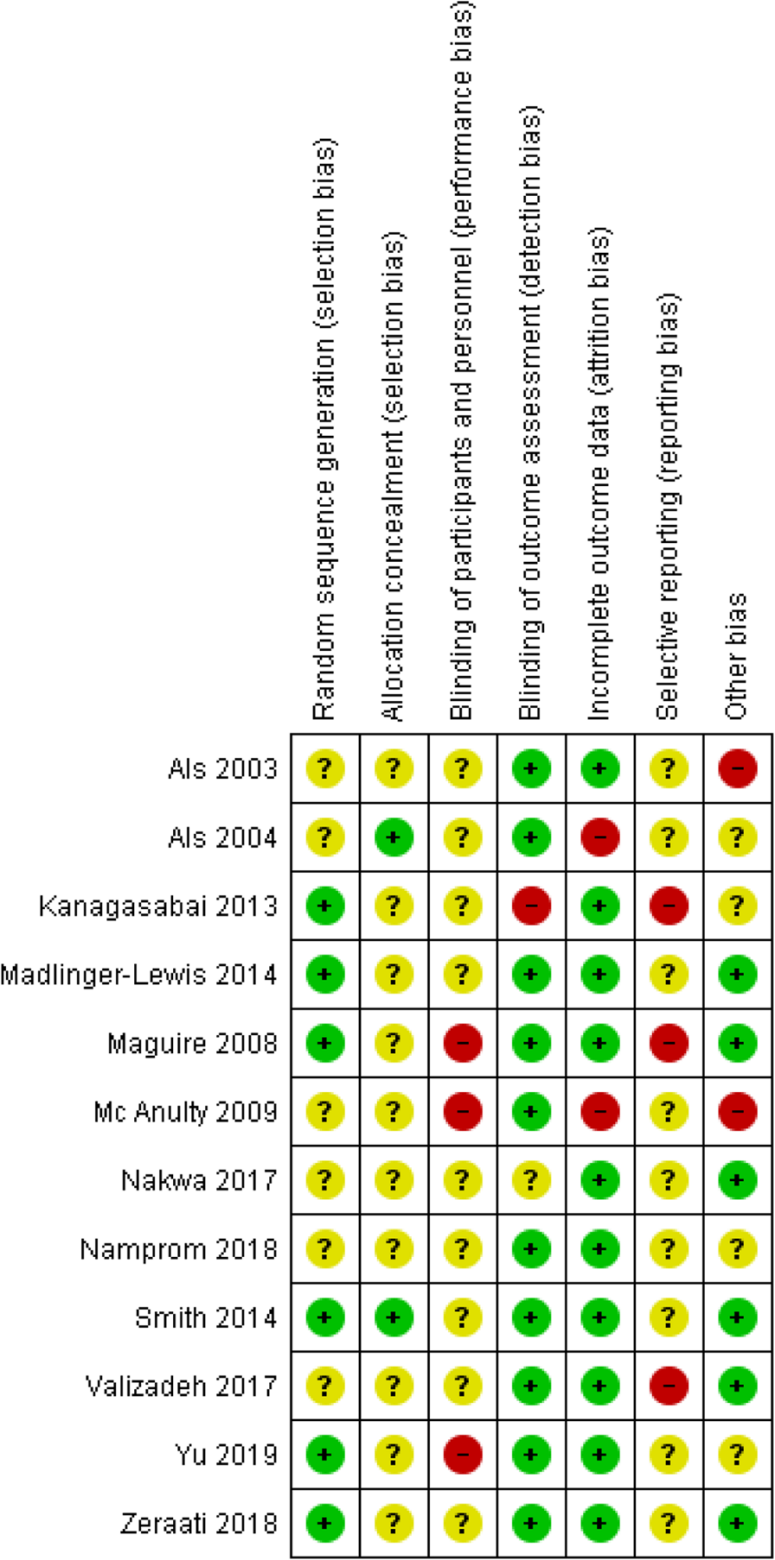
Risk of bias summary: review authors’ judgements about each risk of bias item for each included study

The risk of bias assessment graph for the included studies is presented in Additional File 3 – Figure S1 (details for each included study); a summarization figure in Fig. 2 and a summary table in Additional File 2 – Table S2 are also presented. Six of the 12 studies reported the random-sequence generation appropriately [25, 27, 33, 36, 38, 39], while the process used was not clearly indicated in the other studies. Only two studies adequately describe their allocation concealment method [32, 36]. The risk of bias from the blinding of personnel was high in three studies [25, 34, 38] due to the nature of the intervention, and it was unclear for all the other studies because of insufficient information. Blinding of outcome assessment was adequately performed in 10 studies except for one rated high-risk [33] and one unclear for the study not addressing this outcome [35]. All the studies were judged low for incomplete outcome data except for two: one did not provide reasons for missing data [32] and one had an imbalance numbers in groups [34]. Selective reporting was unclear in all studies and this risk of bias was high in three studies because not all prespecified outcomes were reported [33], or a difference was noticed between the protocol registered and the publication [25, 37]. We considered six studies to be free from other sources of bias [25, 27, 35–37, 39], whereas two studies were judged high since an important risk of bias was associated with threats of study validity [24, 34] and four had unclear risk of bias for insufficient rationale or evidence provided [26, 32, 33, 38] (see Additional File 3 – Table S2 for a detailed explanation).

### Risk of bias across studies

As no more than ten studies were included in the meta-analysis, funnel plot asymmetry was not tested, as the power of this test would be too low to distinguish an asymmetry indicating a publication bias [28]. However, we performed different strategies to decrease potential reporting bias, including a comprehensive search by an expert librarian using nine different databases, an online search of several trial registries to identify relevant published trials and contacting authors by email to obtain missing data.

### Synthesis of results

#### Developmental care vs. Standard care

##### NIDCAP. Neurobehavioral development

**Fig. 3:**
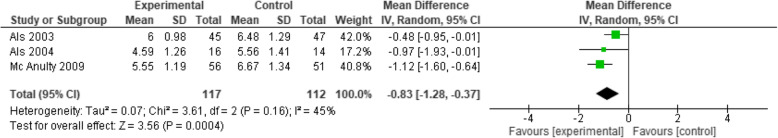
NIDCAP vs. Standard care for the neurobehavioral development (autonomic system - APIB)

**Fig. 4:**
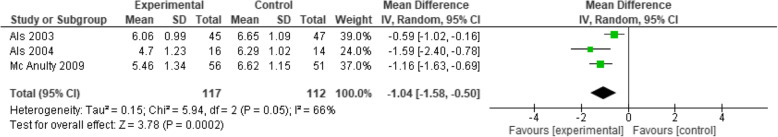
NIDCAP vs. Standard care for the neurobehavioral development (motor system - APIB)

Three studies [24, 32, 34] that included a total of 229 participants (treatment: *n* = 117, control: *n* = 112) investigated the effects of NIDCAP compared to standard care using the APIB scale. Compared to standard care, the effect of NIDCAP was found to significantly improve preterm infants’ autonomic system (MD -0.83; 95% CI − 1.28 to − 0.37; I^2^ = 45%; *p* = 0.0004) (see Fig. 3), motor system (MD -1.04; 95% CI − 1.58 to − 0.50; I^2^ = 66%; *p* = 0.0002) (see Fig. 4), state system (MD -0.74; 95% CI − 1.06 to − 0.42; I^2^ = 0%; *p* < 0.00001) (Additional File 4 – Figure S2), interaction-attentional system (MD -0.48; 95% CI − 0.85 to − 0.11; I^2^ = 0%; *p* = 0.01) (Additional File 5 – Figure S3), and self-regulatory system (MD -0.84; 95% CI − 1.17 to − 0.51; I^2^ = 9%; *p* < 0.00001) (Additional File 6 – Figure S4). The effect of NIDCAP also significantly improved the examiner facilitation subscale (MD -1.02; 95% CI − 1.44 to − 0.60; I^2^ = 0%; *p* < 0.00001) (Additional File 7 – Figure S5).

##### NIDCAP. Neurological development

**Fig. 5:**
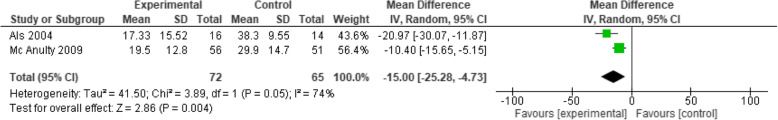
NIDCAP vs. Standard care for neurological development (Prechtl)

Two studies [32, 34] totalling 137 participants (treatment: *n* = 72, control: *n* = 65) investigated the effects of NIDCAP compared to standard care using the Prechtl Neurological Examination of the Full-term Newborn. The NIDCAP was found to significantly improve preterm infants’ neurological development (MD -15.00; 95% CI − 25.28 to − 4.73; I^2^ = 74%; *p* = 0.004) (see Fig. 5).

##### Alternative positioning. Neurobehavioral development

In one study [27], the effect of positioning was evaluated using the NNNS and the preterm infants in the treatment group showed significantly less asymmetry than those in the control group. Only one significant effect was reported for the asymmetry subscale (MD 0.88; 95% CI 0.45–1.31; *p* < 0.0001), while no significant effect was found for the other NNNS subscales (i.e., attention, handling, quality of movement, regulation, nonoptimal reflexes, stress abstinence, arousal, hypotonicity, hypertonicity, excitability and lethargy).

##### Positioning and incubator covers. Neurological development

Only one study [25] with 148 participants (treatment: *n* = 76, control: n = 72) investigated the effects of incubator covers and positioning compared to standard care on preterm infants using the Prechtl Neurological Examination of the Full-term Newborn (normal vs. abnormal). No significant effect between groups was found (RR 0.93; 95% CI 0.70 to 1.22; *p* = 0.58).

#### Parental participation intervention vs. Standard care

##### Neurobehavioral development

**Fig. 6:**
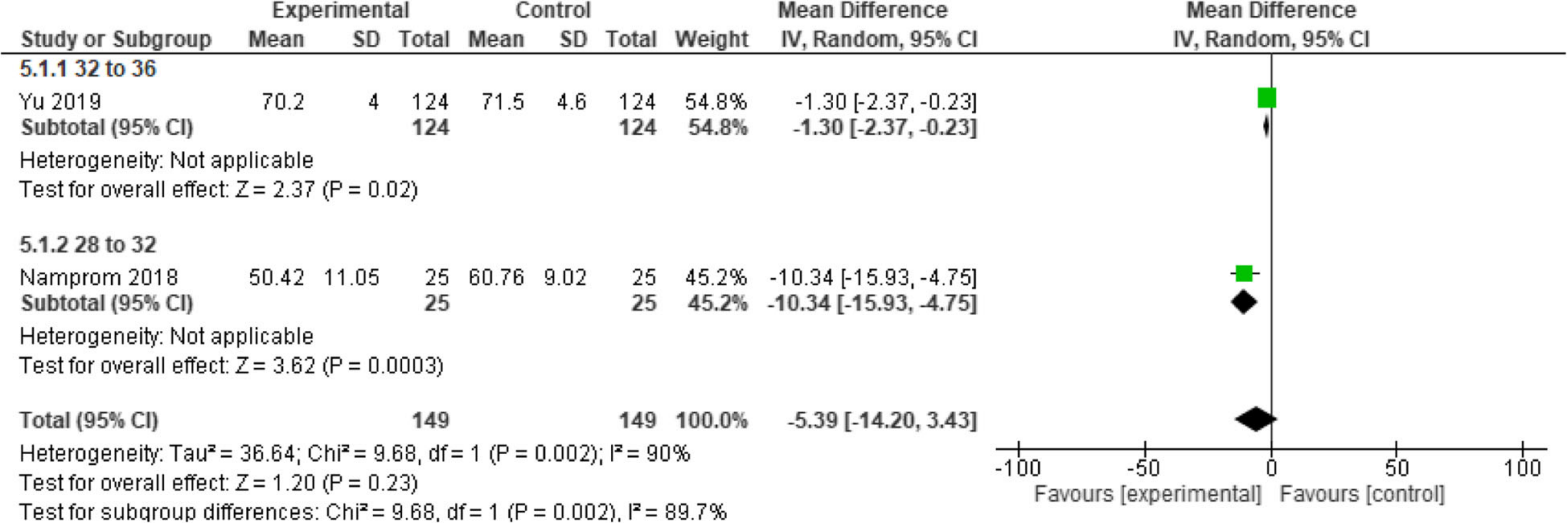
Parental participation programs vs. Standard care forn eurobehavioral development (NNE)

Two studies [26, 38] that included 294 participants (treatment: *n* = 145, control: *n* = 149) investigated the effects of a parental participation program compared to standard care on preterm infants using the NNE. Compared to standard care, the program was not found to significantly improve neurobehavioral development (MD 5.39; 95% CI − 3.43 to 14.20; I^2^ = 90%; *p* = 0.23) (see Fig. 6).

#### Sensory stimulation vs. Standard care

##### Tactile. Neurobehavioral development

One study [36] that included 18 participants (treatment: *n* = 9, control: n = 9) investigated the effects of a tactile intervention using the NNNS. No significant difference between groups was found for any of the 12 subscales (i.e., attention, handling, quality of movement, regulation, nonoptimal reflexes, asymmetric reflexes, stress abstinence, arousal, hypotonicity, hypertonicity, excitability and lethargy).

##### Multisensory. Neuromotor development

Only one study [33] that included 50 participants (treatment: *n* = 25, control: n = 25) investigated the effects of a multisensory stimulation intervention compared to standard care, assessed with the INFANIB. The multisensory stimulation was significantly in favour of the experimental group (MD 3.08; 95% CI 1.33–4.83; *p* = 0.0005).

##### Multisensory. Neuromuscular Development

One study [39] with 80 participants (treatment: *n* = 40; control: n = 40) evaluated the effects of a multisensory intervention compared to standard care using the New Ballard score. Both groups showed significant improvement before and after, but infants of the experimental group had significantly higher neuromuscular development after the intervention compared to infants of the comparator group (MD 5.60; 95% CI 4.65–6.55; *p* < 0.00001).

#### Music vs. Developmental care

##### Neuromotor and neuromotor development

In one study [35] that included 36 participants (treatment: *n* = 18, control: n = 18), the effect of music compared to developmental care was evaluated using the TIMPS and the INFANIB. Significant effects of music were reported for infants’ neuromotor development measured with the TIMPS (MD 0.39; 95% CI 0.08–0.70; *p* = 0.01) and the INFANIB (MD 1.89; 95% CI 0.42–3.36; *p* = 0.01) compared the control group.

#### Physical activity and/or hydrotherapy vs. Containment

##### Neuromotor and neuromuscular development

One study [37] of 38 preterm infants (treatment: *n* = 19, control: n = 19) investigated the effects on neuromotor and neuromuscular development of three different interventions – physical activity, hydrotherapy and a combination of physical therapy and hydrotherapy – compared to containment, using the TIMP, the New Ballard score and items from the Dubowitz examination. For all interventions, the ANOVA effects were not significant: physical therapy (mean: 50.21) vs. containment (mean: 51.57); hydrotherapy (mean 48.05) vs. containment (mean 51.57); or physical therapy combined with hydrotherapy (mean: 52.00) vs. containment (mean: 51.57): *p* = 0.11. For the neuromuscular development, no significant findings were found for the New Ballard score (*p* > 0.05) while for the two items of the Dubowitz, ankle dorsiflexion was not significantly different between groups, but leg recoil was significantly better for the physical therapy and hydrotherapy groups (*p* = 0.04).

### Quality of evidence

The overall quality of evidence was considered low to very low. The summary findings table is presented by outcome (see Additional File 8 – Table S3). For the comparison between NIDCAP and standard care, the overall quality of evidence was rated low to very low for the autonomic system, motor system, state system, interaction-attention system, self-regulatory systems and examiner facilitation (neurobehavioral development), and very low for neurological development. For the comparison between parental participation program and standard care, the quality of evidence was rated very low. The main reasons for downgrading scores were high risk of bias, high heterogeneity between studies and small sample sizes. For the other comparisons including only one study, the summary of findings table is reported for each outcome (see Additional File 8 – Table S3).

## Discussion

### Summary of evidence

To our knowledge, this is the first systematic review examining the effectiveness of interventions on preterm infants’ neurodevelopment during NICU hospitalization or close to term CA. Our review synthesized the findings of 12 studies that included 901 preterm infants. In our systematic review, we combined the studies which had the same/similar interventions. Combining studies for a meta-analysis was only possible for NIDCAP and programs involving parents. For all other studies included, those were not combined for analysis and results were only described individually in a narrative form. We combined three studies [24, 32, 34] in a meta-analysis showing the positive effects of the NIDCAP intervention compared to standard care on preterm infants’ neurobehavioral and neurological development at two weeks CA. We also combined two other studies [26, 38] in a meta-analysis indicating that, compared to standard care, parental participation interventions do not improve preterm infants’ neurobehavioral development during NICU hospitalization.

For all other interventions, the synthesis shows that compared to standard care or other type of comparators, the effectiveness was either controversial or partial, as significant findings were only reported for one or some subscales of the instruments used to assess the preterm infants’ neurodevelopment. Overall, multisensory stimulation interventions were found to improve neuromuscular development [39] and neurological development [33]. In addition, music was reported to improve neuromotor and neurological development [38]. Conversely, a tactile stimulation intervention along with physical activity and/or hydrotherapy was not found to improve neurobehavioral [36] or neuromotor and neuromuscular development [37]. Developmental care interventions were not found to improve neurobehavioral [27] or neurological development [25]. It should be noted that these findings are only based on single studies with distinctive differences in the nature and components of the interventions, along with the instruments used to assess neurodevelopment.

### Quality of evidence

The overall quality of evidence of the studies included in this systematic review is low to very low, which may be attributed to many high to unclear risk of bias, heterogeneity and small sample sizes. For the allocation risk of bias, all studies except two [32, 36] were judged unclear, as insufficient details were provided. Also, the risk of bias associated with blinding of personnel and participants was either unclear or high in all studies, whereas the selective reporting bias was rated as unclear or high in all studies. Among the 12 included studies, only three included more than 100 participants [25, 34, 38], suggesting that the majority of the included studies were underpowered to detect effects.

Although the meta-analysis indicated that NIDCAP developmental care intervention improved the neurobehavioral and neurological development of preterm infants, these results are based on low to very low evidence that may specifically be explained by unclear to high risk of biases and the small sample size of the combined studies (*n* = 229). It is also interesting to note in the meta-analysis that only three APIB subscales (i.e., autonomic, motor and examiner facilitation) and the Prechtl Neurological Examination of the Full-term Newborn had moderate to considerable statistical heterogeneity (I^2^ from 45 to 74%), which could be attributed to methodological variability arising from potential differences among the evaluators assessing these outcomes [28]. For the parental participation programs, even though the meta-analysis did not support these interventions as favouring the infants’ neurodevelopment, the quality of evidence was very low and may, in this case, be attributed to significant clinical heterogeneity (I^2^ = 90%), possibly resulting from differences in the components of the interventions and the gestational age of preterm infants targeted in these studies [28].

### Comparisons with previous studies

Previous systematic reviews and meta-analysis were conducted with NIDCAP and developmental care interventions to assess the effectiveness of these interventions on the mid- and long-term neurodevelopmental outcomes of preterm infants [10, 11, 13, 40]. For NIDCAP, the systematic review and meta-analysis concluded that significant findings favoring the NIDCAP were only found at 9 months of age but not at 4-, 12-, 18- or 24-months CA. Other recent systematic reviews reported that developmental care interventions favored long-term neurodevelopmental outcomes in preterm infants with significant effects for cognitive, mental, psychomotor and language development up to 18 months of age and IQ at the age of 5 [10, 11]. Our systematic review adds to the efforts of these systematic reviews by bringing in new evidence and reinforcing that the NIDCAP favors preterm infants’ early neurodevelopment at a time that is crucial for brain development and maturation [2, 3].

### Implications for clinical practice

It is imperative to know which interventions during NICU hospitalization promote optimal preterm infants’ early and long-term neurodevelopmental outcomes. Based on our meta-analysis, the NIDCAP favored preterm infants’ neurobehavioral and neurological development at two weeks CA, and even though the quality of evidence was low to very low, it could be recommended in clinical practice. As recent guidelines recommendations arising from systematic reviews suggest, the NIDCAP is recommended as a support to neuroprotective developmental practice care in NICUs [41]. The NIDCAP requires extensive training and time investment to maintain knowledge and expertise [42]. Although it may not be readily accessible for all NICUs, principles guiding developmental care, as NIDCAP, should still be encouraged in neonatal care as DC interventions are recommended to promote short- and long-term neurodevelopmental outcomes in preterm infants [6].

Parental participation programs were not found to improve preterm infants’ neurobehavioral development and had very low quality of evidence, which may be explained by the different intervention components and the gestational age of preterm infants included in the studies. The diverse role that parents may play in the early NICU participation programs could perhaps have accounted for the high heterogeneity between studies. Still, family-centered care in the NICU is a central developmental care intervention, and interventions fostering parental participation in infant care are recommended for clinical practice [43]. Parental presence, educational sessions and active parental participation in preterm infants’ care have been recently been reported to improve both infant and parental outcomes [44] and should therefore be promoted in NICUs. A summary of the meta-analysis main findings and clinical implications is presented in Figure S6 – see Additional file 9.

### Implications for research

First, a clear conceptual definition of preterm infants’ neurodevelopment should be formulated to guide the choice of interventions as well as the measurement tools used in experimental studies. Future studies could test the different interventions included in this systematic review to build on evidence for those interventions. In addition, analysis according to gender difference could also contribute to knowledge development about the effectiveness of the interventions.

Different instruments were used in our systematic review to evaluate the effectiveness of interventions on preterm infants’ neurodevelopment during NICU hospitalization. Although these instruments all have standardized administration approaches and scoring [45], they measure slightly different aspects of neurodevelopment (i.e., the APIB assesses neurobehavioral development, the Precthl assesses neurological development and the TIMP assesses neuromotor development), limiting comparisons between included studies. Including a combination of different instruments [32, 35, 37, 39] could provide a more global assessment of preterm infants’ neurodevelopment and thus allow for comparisons across studies.

One systematic review on developmental care [10] recommended combining electroencephalography (EEG) to a measure of neurobehavioral development for assessing the effectiveness of interventions with preterm infants. Recent advances support the idea that brain activity and oxygenation during the neonatal period play a crucial role in the preservation and development of brain connections, and thus in infants’ brain functioning and growth [46, 47], and can predict their neurodevelopmental status in early childhood [48, 49]. Therefore, combined with other standardized instruments, EEG as a measure of infants’ neurodevelopment could be used in future studies. Still, EEG as a measure of neurodevelopment needs more research in order to be more objective and therefore more comparable across studies.

For research purposes, a systematic review of neonatal assessments supports the use of the APIB and the NNNS, which have adequate psychometrics qualities [19]. The choice of instruments to measure preterm infants’ neurodevelopment during NICU hospitalization could also be based on its predictive validity for long-term neurodevelopment. The NNNS has predictive validity, as it correlates with the Bayley cognitive scores at 12 or 24 months of age [50]. Of the instruments used in the studies included in our systematic review, the TIMP is the neuromotor assessment with the best predictive validity for long-term neurodevelopmental outcomes [18].

### Limitations

Our review differed from the published protocol with respect to the inclusion criteria and term age: we included neurodevelopment measurements done soon after discharge at two weeks CA. We only included French and English literature, and missing data precluded us from including one study in the meta-analysis for the NICDAP intervention. These differences were clearly reported in the manuscript.

Moreover, a description of the standard of care was provided in 8 of the 12 studies [24, 27, 32, 33, 35–38], but the description was very different among the studies and may reflect the evolution of care in neonatology, since our systematic review included studies conducted 16 years apart. Likewise, the standard of care may have differed based on the NICU design of the included studies, since in recent decades neonatal units have modified their unit configuration to single-family rooms, as this is the recommended NICU design [51, 52]. The ability to operationalize interventions between the different NICU designs could have played a role and other environmental conditions not readily evident may account for some variability. Standard care and interventions conditions in experimental studies should be reported as per the guidelines. Together, these aspects limit the conclusions of our systematic review, and future studies evaluating the effectiveness of interventions are recommended.

## Conclusions

Future studies are needed to identify the interventions that are the most effective in promoting preterm infants’ neurodevelopment during NICU hospitalization or close to term age. Without a clear definition of neurodevelopment, interventions should be appropriately designed to allow comparison with previous studies and instruments should be combined to measure different aspects of infants’ neurodevelopment. NICU hospitalization is a critical period for the brain development of preterm infants, and all experiences encountered can significantly shape their neurodevelopment, so it is imperative to identify which interventions in the NICU optimize short-term health outcomes in preterm infants.

## Supplementary Information

**Additional file 1: Table S1**. Search strategy in MEDLINE – table presenting the search strategy with keywords and results.

**Additional file 2: Table S2.** Bias summary of included tudies – table presenting the judgement of the Cochrane’s risk of bias tool for each individual study.

**Additional file 3: Figure S1**. Risk of bias summary: review authors’ judgements about each risk of bias item presented as percentages across all included studies.

**Additional file 4: Figure S2**. NIDCAP vs. Standard care for the neurobehavioral development (state system - APIB) – figure presenting a meta-analysis.

**Additional file 5: Figure S3**. NIDCAP vs. Standard care for the neurobehavioral development (attention-interaction system - APIB) - figure presenting a meta-analysis.

**Additional file 6: Figure S4**. NIDCAP vs. Standard care for the neurobehavioral development (self-regulation system - APIB) - figure presenting a meta-analysis.

**Additional file 7: Figure S5**. NIDCAP vs. Standard care for the n eurobehavioral development (examiner facilitation - APIB) - figure presenting a meta-analysis.

**Additional file 8: Table S3.** Quality of evidence of studies included in meta-analysis – table presenting the quality of evidence according to the GRADE tool for each intervention and outcomes.

**Additional file 9: Figure S6**. Summary of meta-analysis main findings and clinical implications.

## Data Availability

All data generated or analysed during this study are included in this published article and its supplementary information files.

## Abbreviations

BW: Birthweight
APIB: Assessment of Preterm Infants’ Behavior
CA: corrected age
CI: Confidence Interval
EEG: Electroencephalogram
GRADE: Grading of Recommendations Assessment, Development and Evaluation working group methodology
INFANIB: Infant Neurological International Battery
IVH: Intraventricular Hemorrhage
NIDCAP: Newborn Individualized Developmental Care and Assessment Program
NICU: Neonatal Intensive Care Unit
NNE: Neonatal Neurobehavioral Examination
NNNS: NICU Network Neurobehavioral Scale
PRISMA: Preferred Reporting Items for Systematic review and Meta-Analysis
RCT: Randomized Controlled Trial
MD: Mean Difference
TIMP: Test of Infant Motor Performance
WGA: Weeks Gestational Age
