# Supplementary material for: Effectiveness of interventions on early neurodevelopment of preterm infants: a systematic review and meta-analysis
Source: BMC Pediatr. 2021 Apr 29;21:210. doi: 10.1186/s12887-021-02559-6 (PMC8082967; doi:10.1186/s12887-021-02559-6)
Supplement: Supplementary file 1 — Additional file 1: Table S1. Search strategy in MEDLINE – table presenting the search strategy with keywords and results. [file 12887_2021_2559_MOESM1_ESM.docx]

**Table S1**. Search strategy in MEDLINE

MEDLINE

|  | **Search strategy** | **Results** |
| --- | --- | --- |
| 1 | exp intensive care units, pediatric/ or Neonatal Nursing/ or Intensive Care, Neonatal/ or intensive care units/ or hospital units/ or nurseries, hospital/ or exp pediatrics/ | 127 964 |
| 2 | (((neonat* OR pediatric$1 OR birth$1 OR newborn* OR bab$3 OR infant$1) adj5 ("intensive care?" OR "care unit?" OR "care center?" OR hospital*)) OR "NICU" OR "perinatal center?" OR "growing care unit?" OR "intensive care unit?").ti,ab | 120 198 |
| 3 | neurodevelopmental disorders/ or Neurologic Examination/ or psychomotor performance/ or neurobehavioral manifestations/ or psychomotor disorders/ or cognition disorders/ or auditory perceptual disorders/ or cognitive dysfunction/ or Child Development/ or motor skills/ or motor skills disorders/ or Motor Activity/ or Movement Disorders/ | 302 922 |
| 4 | (neurodevelopment* OR ((neurologic* OR neurobehavi* OR neuromotor* OR cogniti* OR cerebral OR brain? OR "nervous system") adj3 (problem$1 OR impair* OR abilit$3 OR disorder$1 OR disab* OR development*)) OR (motor adj3 (skill$1 OR activit$3 OR control OR impair*)) OR "infant development").ti,ab | 231 230 |
| 5 | exp infant, premature/ or exp infant, low birth weight/ or premature birth/ or *Infant, Newborn/ | 90 858 |
| 6 | ((infant$1 OR bab$3 OR newborn$1 OR birth$1 OR neonat*) adj4 (prematur* OR pre?term OR (low adj2 weight))).ti,ab | 82 130 |
| 7 | (1 OR 2) AND (3 OR 4) AND (5 OR 6) | 1 916 |
| 8 | 7 AND (English OR French).lg | 1 775 |
