# Supplementary material for: Effectiveness of interventions on early neurodevelopment of preterm infants: a systematic review and meta-analysis
Source: BMC Pediatr. 2021 Apr 29;21:210. doi: 10.1186/s12887-021-02559-6 (PMC8082967; doi:10.1186/s12887-021-02559-6)
Supplement: Supplementary file 2 — Additional file 2: Table S2. Bias summary of included tudies – table presenting the judgement of the Cochrane’s risk of bias tool for each individual study. [file 12887_2021_2559_MOESM2_ESM.docx]

| **Table S2.** Bias Summary of Included Studies | | | | | | | |
| --- | --- | --- | --- | --- | --- | --- | --- |
| **Studies** | **Randomization (random sequence generation)** | **Allocation bias** | **Blinding of participants and personnel** | **Blinding of outcome assessment** | **Incomplete outcome data** | **Selective outcome reporting** | **Other potential sources of bias** |
| **Als 2003** | **Unclear risk of bias** | **Unclear risk of bias** | **Unclear risk of bias** | **Low risk of bias** | **Low risk of bias** | **Unclear risk of bias** | **High risk of bias** |
|  | Insufficient information about the sequence generation process to permit judgement of ‘Low risk’ or ‘High risk’. | Insufficient information to permit judgement of ‘Low risk’ or ‘High risk’. | The study did not address this outcome.  Participants were not blinded but we don’t know about the blinding of the personnel for the control group. | “*All outcome assessments were conducted by trained examiners blind to the group membership of the infants and parents and to the specific goals of the study*.”  Blinding of outcome assessment ensured, and unlikely that the blinding could have been broken. | *“Of the 110 infants successfully enrolled, 18 died (BWH, 10; CHO, 8; CHB, 0)* *resulting in 92 study infants…*”  Missing outcome data balanced in numbers across intervention groups, with similar reasons for missing data across groups. | The study protocol not published, and study not registered. | “…*the CHO NICU was significantly brighter, louder, and more active than the BWH or the CHB*.”  “*Children’s Hospital Oakland (CHO), the nursery with the lowest developmental care scores, the socioculturally most challenged families, and the initially sickest infants,achieved the largest shift in developmental outcome*”.  There is at least one important risk of bias related to study settings. |
| **Als 2004** | **Unclear risk of bias** | **Low risk of bias** | **Unclear risk of bias** | **Low risk of bias** | **High risk of bias** | **Unclear risk of bias** | **Unclear risk of bias** |
|  | “*A controlled trial design with 2-group randomization was used. Blocking by gender (male/f emale) and ethnicity (white/ ther) was imposed a priori*.”  The investigators describe a random component in the sequence generation process but unsure if the randomization box could influence the random sequence generation. | “*Group assignment was revealed by parental opening of the opaque, prenumbered, sealed envelope drawn from the respective randomization box*.”  Participants and investigators enrolling participants could not foresee assignment because one of the following, or an equivalent method, was used to conceal allocation: Sequentially numbered, opaque, sealed envelopes. | Insufficient information to permit judgment of ‘Low risk’ or ‘High risk’. | “*Outcome assessment staff was purposefully kept “blind” to the infants’ group assignments.*”  Blinding of outcome assessment ensured, and unlikely that the blinding could have been broken. | No reasons for missing data provided. | The study protocol not published, and study not registered. | “*An additional limitation of the MRI data are the investigator-dependent, operator-driven delineation of ROI chosen for analysis, which potentially might enter a degree of subjectivity into the data analysis*.”  “*No formal effort was made to prevent spillover and contamination effects from experimental to control group care*.”  There may be a risk of bias but insufficient rationale or evidence that an identified problem will introduce bias. |
| **Kanagasabai 2013** | **Low risk of bias** | **Unclear risk of bias** | **Unclear risk of bias** | **High risk of bias** | **Low risk of bias** | **High risk of bias** | **Unclear risk of bias** |
|  | “*Fifty preterm infants were equally block randomized into study and control group (5 blocks of 10 infants each using lottery method*).”  The investigators describe a random component in the sequence generation process such as: Drawing of lots. | Insufficient information to permit judgement of ‘Low risk’ or ‘High risk’. | Insufficient information to permit judgement of ‘Low risk’ or ‘High risk’. | “…*blinding of the assessor was not possible owing to feasibility issues such as availability of personnel and time constraints associated with the research*.”  No blinding, and the outcome is likely to be influenced by lack of blinding. | No missing outcome data. | The study protocol not published, and study not registered.  Information about physiological parameters indicated as an outcome but not discussed in results/discussion.  Not all of the study’s pre-specified outcomes have been reported. | “*The average number of intervention session was 12.”*  Insufficient information to assess whether an important risk of bias exists as it appears that not all participants received the same number of interventions. |
| **Madlinger-Lewis**  **2014** | **Low risk of bias** | **Unclear risk of bias** | **Unclear risk of bias** | **Low risk of bias** | **Low risk of bias** | **Unclear risk of bias** | **Low risk of bias** |
|  | “*Participants were randomly assigned following simple randomization procedures (computerized random numbers), which assigned infants to 1 of 2 treatment arms (alternative or traditional positioning methods)”.*  The investigators describe a random component in the sequence generation process such as: Using a computer random number generator. | “*Prior to the first participant being enrolled, the randomization assignment was determined and indicated in a sealed envelope*.”  Insufficient information to permit judgement of ‘Low risk’or ‘High risk’ as it remains unclear whether envelopes were sequentially numbered, opaque and sealed. | The study did not address this outcome. | *“Between 35-40 weeks PMA, infants underwent neurobehavioral testing using the NICU Network Neurobehavioral Scale (NNNS) by a single, certified blinded rater.”*  Blinding of outcome assessment ensured, and unlikely that the blinding could have been broken. | “*Of the 100 infants enrolled, 4 expired and 4 withdrew, leaving 92 infants*.”  Missing outcome data balanced in numbers across intervention groups, with similar reasons for missing data across groups. | The study protocol not published, and study not registered. | The study appears to be free of other sources of bias. |
| **Maguire**  **2008** | **Low risk of bias** | **Unclear risk of bias** | **High risk of bias** | **Low risk of bias** | **Low risk of bias** | **High risk of bias** | **Low risk of bias** |
|  | “*…the DC group or the control group using sealed envelopes made in groups of 6 using a computer-generated randomization allocation*.”  The investigators describe a random component in the sequence generation process such as: Shuffling cards or envelopes. | The use of assignment envelopes is described, but it remains unclear whether envelopes were sequentially numbered, opaque and sealed. | *“…there could be no blinding of the intervention because the infants in the DC group had incubator covers and nesting*.”  No blinding or incomplete blinding, and the outcome is likely to be influenced by lack of blinding. | *“…the neonatologists who performed the term age assessments were blinded to group participation*.”  Blinding of outcome assessment ensured, and unlikely that the blinding could have been broken. | Missing outcome data balanced in numbers across intervention  groups, with similar reasons for missing data across groups. | The study protocol not published, but study was registered prospectively: Trial NL221 (NTR258).  Number of days of intensive care and length of hospitalization were not reported as primary outcome in protocol but are reported in the article.  Although cited as primary outcomes in protocol, no data were reported on Parental Stress Scale-NICU (PSS-NICU), The Nurse Parent Support Tool (NPST) and Mothers and Babies Scale (MABS). | The study appears to be free of other sources of bias. |
| **McAnulty 2009** | **Unclear risk of bias** | **Unclear risk of bias** | **High risk of bias** | **Low risk of bias** | **High risk of bias** | **Unclear risk of bias** | **High risk of bias** |
|  | “*Following parental consent subjects were randomly assigned to the C- or E-group*.”  Insufficient information about the sequence generation process to permit judgement of ‘Low risk’ or ‘High risk’. | Insufficient information about the sequence generation process to permit judgement of ‘Low risk’ or ‘High risk’. | “*Control-group infants received NICU care as standard at the time of study without attempts to influence staffing or prevent spillover from E-group to C-group care*.”  “*Treatment contamination of standard care by NIDCAP appeared to become evident only by the third phase of the study*.”  No blinding or incomplete blinding, and the outcome is likely to be influenced by lack of blinding. | “*…blinded assessors evaluated outcome at 2 wCA and 9mCA.”*  Blinding of outcome assessment ensured, and unlikely that the blinding could have been broken. | “*At 2 wCA, all 107 infants (51C, 56E) had complete neurobehavioural data and 82 (35C, 47E) had complete neurophysiologi-cal data*.”  Reason for missing outcome data likely to be related to true outcome, with either imbalance in numbers or  reasons for missing data across intervention groups. | The study protocol not published, and study not registered. | “*While medical care changed over the 8‐year interval, the study involved time‐matched controls, which assured that the changes did not alter the impact of the intervention.*  *“However, despite contamination, the overall impact of the intervention was still highly significant*.”  There is at least one important risk of bias which may be related to bias historical/bias contamination. |
| **Nakwa**  **2007** | **Unclear risk of bias** | **Unclear risk of bias** | **Unclear risk of bias** | **Unclear risk of bias** | **Low risk of bias** | **Unclear risk of bias** | **Low risk of bias** |
|  | Insufficient information about the sequence generation process to permit judgement of Low risk or High risk.  Authors only report a random sampling method. | Insufficient information to permit judgement of ‘Low risk’ or ‘High risk’. The method of concealment is not described or not described in sufficient detail to allowa definite judgement.  Authors mentioned an explicitly unconcealed procedure - that they separated the groups in 2: group A which was control and group B: experimental. | The study did not address this outcome. | The study did not address this outcome. | *“There were 2 drop outs in each group because of early discharge, thus each group had 18 participants.”*  There were 2 dropouts in each group because of early discharge, thus each group had 18 participants. | The study protocol not published, and study not registered. | The study appears to be free of other sources of bias. |
| **Namprom**  **2017** | **Unclear risk of bias** | **Unclear risk of bias** | **Unclear risk of bias** | **Low risk of bias** | **Low risk of bias** | **Unclear risk of bias** | **Unclear risk of bias** |
|  | Insufficient information about the sequence generation process to permit judgement of Low risk or High risk. | The method of concealment is not described or not described in sufficient detail to allow a definite judgement. | *“The activities were performed by the researcher who is an NICU nurse.”*  Unclear if the nurse was aware of the research goal and unclear information as to whether mothers were blinded to intervention.  Insufficient information to permit judgment of ‘Low risk’ or ‘High risk’. | “*The study groups were blinded to the research assessor and statistical analyst*.”  Blinding of outcome assessment ensured, and unlikely that the blinding could have been broken. | Missing outcome data balanced in numbers across intervention groups, with similar reasons for missing data across groups. | The study protocol not published, and study not registered. | “*Some mothers had no chance to perform activities such as tucking, and kangaroo care while infants were receiving painful procedures. This is because of the hospital visiting policy which does not allow mothers to be with their infants at bedside all day*.”  There may be a risk of bias, but insufficient rationale or evidence that an identified problem will introduce bias. |
| **Smith**  **2014** | **Low risk of bias** | **Low risk of bias** | **Unclear risk of bias** | **Low risk of bias** | **Low risk of bias** | **Unclear risk of bias** | **Low risk of bias** |
|  | *“The randomization process used sequentially numbered, opaque, sealed envelopes that contained an index card with the word treatment or control.”*  The investigators describe a random component in the sequence generation process such as: Shuffling cards or envelopes. | *“Parents were provided the opportunity to select one of the envelopes that determined the infant's allocation.”*  Participants and investigators enrolling participants could not foresee assignment because one of the following, or an equivalent method, was used to conceal allocation: Sequentially numbered, opaque, sealed envelopes. | The study did not address this outcome. | *“All evaluations*  *were performed by a single trained and certified*  *occupational therapist with expertise in evaluating*  *very preterm infant neurobehavior and who was blinded to subject allocation.”*  Blinding of outcome assessment ensured, and unlikely that the blinding could have been broken. | Reasons for missing outcome data unlikely to be related to true outcome (for survival data, censoring unlikely to be introducing bias). | The study protocol not published, and study not registered. | The study appears to be free of other sources of bias. |
| **Valizadeh 2017** | **Unclear risk of bias** | **Unclear risk of bias** | **Unclear risk of bias** | **Low risk of bias** | **Low risk of bias** | **High risk of bias** | **Low risk of bias** |
|  | *“The statistical analysis and randomization were done by an associate professor of statistics who was blinded and independent from the study*.”  Insufficient information about the sequence generation process to permit judgement of ‘Low risk’ or ‘High risk’. | *“The infants were allocated to physical activity, hydrotherapy, combination, and containment groups through random blocks of sizes 4 and 8*.”  Insufficient information to permit judgement of ‘Low risk’ or ‘High risk’. | The study did not address this outcome. | *“During the assessment, both the examiners and the statistical analysis were blind about the type of intervention received by the infants.”*  Blinding of outcome assessment ensured, and unlikely that the blinding could have been broken. | Reasons for missing outcome data unlikely to be related to true outcome (for survival data, censoring unlikely to be introducing bias). | The study protocol not published, but study was registered in the Iran Registry of Clinical Trials (IRTC) (code: N7201405208315).  Although cited as primary outcomes in protocol, no data were reported on physiological and behavioral data (APIB & pulse oximetry). | The study appears to be free of other sources of bias. |
| **Yu**  **(2017)** | **Low risk of bias** | **Unclear risk of bias** | **High risk of bias** | **Low risk of bias** | **Low risk of bias** | **Unclear risk of bias** | **Unclear risk of bias** |
|  | *“Infants were randomly allocated into groups receiving FCIP and the usual care program (UCP) according to a computer-generated random sequence stratified by hospital, sex, gestational age, and maternal education level (detailed procedure described elsewhere)”.*  The investigators describe a random component in the sequence generation process such as: Using a computer random number generator. | Insufficient information to permit judgement of ‘Low risk’ or ‘High risk’. | *“… the intervention providers and parents were aware of the group allocations”.*  No blinding or incomplete blinding, and the outcome is likely to be influenced by lack of blinding. | “*The neonatologists, nursing staff, and*  *outcome assessors were masked with regard to the group*  *assignments of the infants,…”.*  Blinding of outcome assessment ensured, and unlikely that the blinding could have been broken. | *“…,18*  *did not receive their allocated interventions because they*  *dropped out, expired, or terminated their participation*  *early, leaving 122 FCIP group infants and 129 UCP group*  *infants for intervention and follow-up.”*  Infants not examined in the intervention group because of prolonged crying (n=1) and being on CPAP (n=1) and in the control group for parental inconvenience (n=2) and being on CPAP (n=3).  Missing outcome data balanced in numbers across intervention groups, with similar reasons for missing data across groups. | This study protocol is registered at ClinicalTrials.gov (NCT01807533).  Study is reporting only 2 prespecified secondary outcomes. | There were a few infants (1 in the FCIP group and 3 in the UCP group) whose parents requested a change of group allocation because of personal preference or time constraints; their data were analyzed according to the original group allocations.  There may be a risk of bias, but insufficient information to assess whether an important risk of bias exist. |
| **Zeerati**  **(2019)** | **Low risk of bias** | **Unclear risk of bias** | **Unclear risk of bias** | **Low risk of bias** | **Low risk of bias** | **Unclear risk of bias** | **Low risk of bias** |
|  | *“…80 preterm were randomly enrolled in either intervention and control groups (40 infants in each group) using random number table. They had an equal probability of being assigned to each of the two groups. »*  The investigators describe a random component in the sequence generation process such as: Referring to a random number table. | Insufficient information to permit judgement of ‘Low risk’or ‘High risk’. | The study did not address this outcome. | *“In order to lack of bias in the results of the study, who measure to assess the neuromotor development of the preterm by using the New Ballard Scale was blind to the groups.”*  Blinding of outcome assessment ensured, and unlikely that the blinding could have been broken. | No missing outcome data reported. | The study protocol is registered (ID: IRCT2016073114454N2).  Study is reporting only 2 prespecified secondary outcomes. | The study appears to be free of other sources of bias. |

Note: Italics shows direct quotes from articles.
