# Supplementary material for: Effectiveness of interventions on early neurodevelopment of preterm infants: a systematic review and meta-analysis
Source: BMC Pediatr. 2021 Apr 29;21:210. doi: 10.1186/s12887-021-02559-6 (PMC8082967; doi:10.1186/s12887-021-02559-6)
Supplement: Supplementary file 3 — Additional file 3: Figure S1. Risk of bias summary: review authors’ judgements about each risk of bias item presented as percentages across all included studies. [file 12887_2021_2559_MOESM3_ESM.docx]

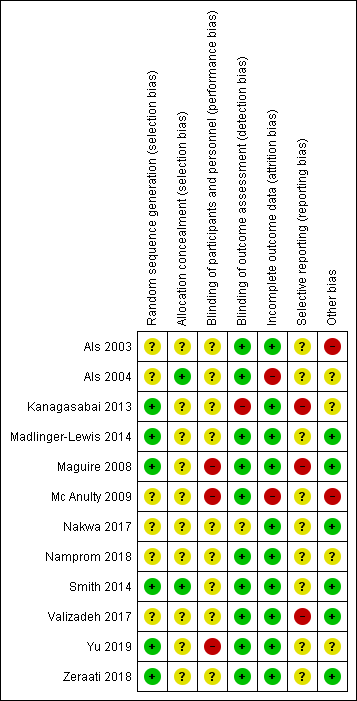


**Figure S1**. Risk of bias summary: review authors' judgements about each risk of bias item for each included study.
