# Supplementary figures and images for: Effectiveness of interventions on early neurodevelopment of preterm infants: a systematic review and meta-analysis
Source: BMC Pediatr. 2021 Apr 29;21:210. doi: 10.1186/s12887-021-02559-6 (PMC8082967; doi:10.1186/s12887-021-02559-6)

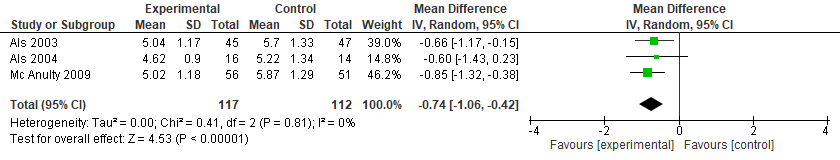


**Figure S2**. NIDCAP vs. Standard Care for the Neurobehavioral Development (state system - APIB)

Supplement: Supplementary file 4 — Additional file 4: Figure S2. NIDCAP vs. Standard care for the neurobehavioral development (state system - APIB) – figure presenting a meta-analysis. [file 12887_2021_2559_MOESM4_ESM.docx]

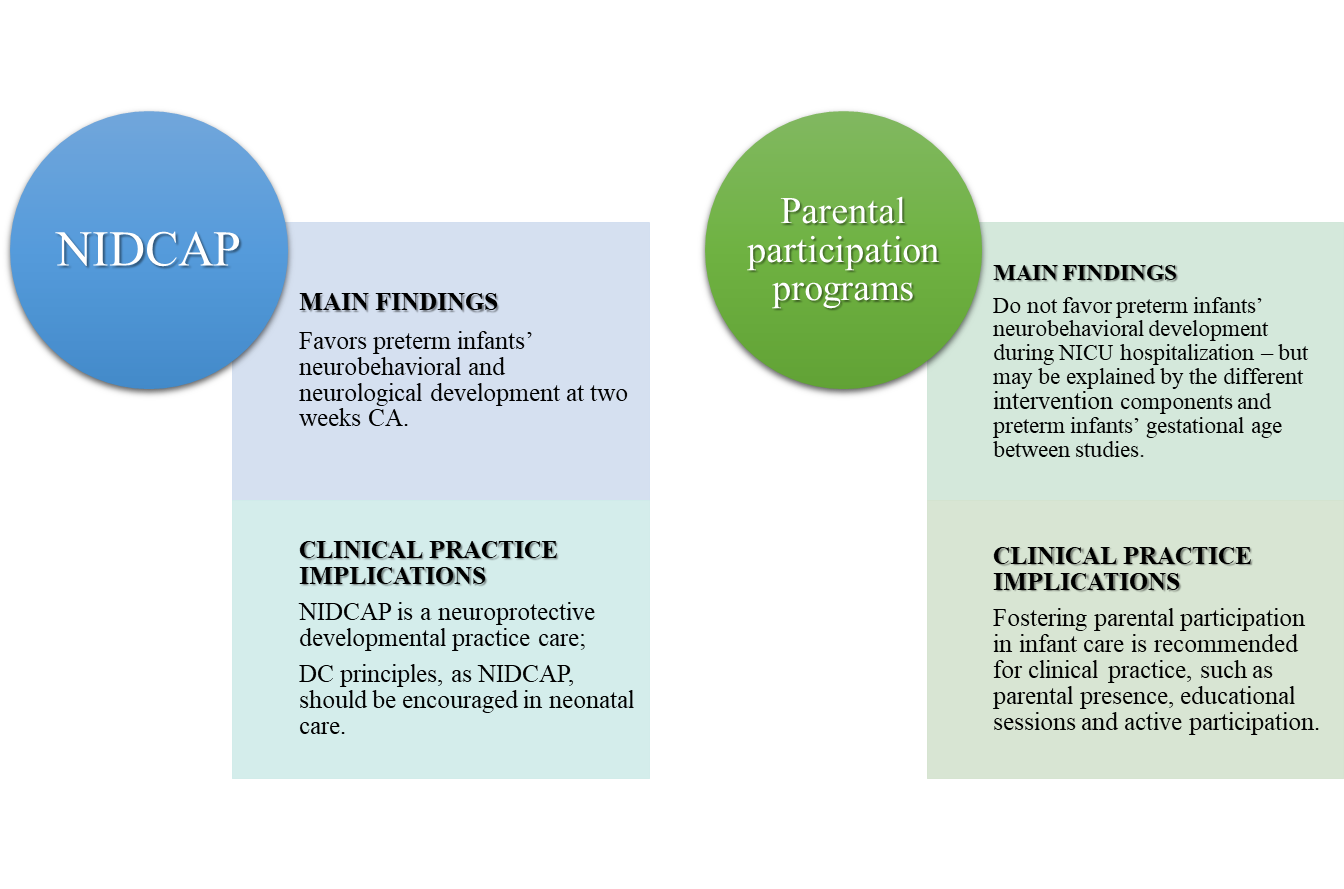


**Figure 6S**. Summary of meta-analysis main findings and clinical implications.

Supplement: Supplementary file 9 — Additional file 9: Figure S6. Summary of meta-analysis main findings and clinical implications. [file 12887_2021_2559_MOESM9_ESM.docx]
