# Supplementary material for: Effectiveness of interventions on early neurodevelopment of preterm infants: a systematic review and meta-analysis
Source: BMC Pediatr. 2021 Apr 29;21:210. doi: 10.1186/s12887-021-02559-6 (PMC8082967; doi:10.1186/s12887-021-02559-6)
Supplement: Supplementary file 5 — Additional file 5: Figure S3. NIDCAP vs. Standard care for the neurobehavioral development (attention-interaction system - APIB) - figure presenting a meta-analysis. [file 12887_2021_2559_MOESM5_ESM.docx]

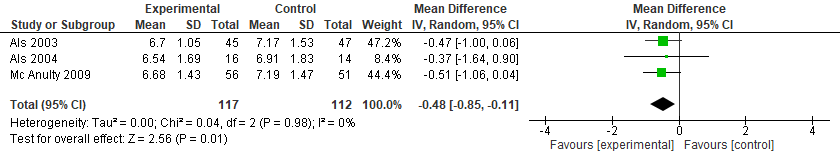


**Figure 3S**. NIDCAP vs. Standard Care for the Neurobehavioral Development (attention- interaction system - APIB)
