# Supplementary material for: Effectiveness of interventions on early neurodevelopment of preterm infants: a systematic review and meta-analysis
Source: BMC Pediatr. 2021 Apr 29;21:210. doi: 10.1186/s12887-021-02559-6 (PMC8082967; doi:10.1186/s12887-021-02559-6)
Supplement: Supplementary file 8 — Additional file 8: Table S3. Quality of evidence of studies included in meta-analysis – table presenting the quality of evidence according to the GRADE tool for each intervention and outcomes. [file 12887_2021_2559_MOESM8_ESM.docx]

**Table S3.** Quality of Evidence of studies

Developmental Care - NIDCAP compared to Standard care

| **Certainty assessment** | | | | | | | **№ of patients** | | **Effect** | | **Certainty** |
| --- | --- | --- | --- | --- | --- | --- | --- | --- | --- | --- | --- |
| **№ of studies** | **Study design** | **Risk of bias** | **Inconsistency** | **Indirectness** | **Imprecision** | **Other considerations** | **NIDCAP** | **Standard care** | **Relative (95% CI)** | **Absolute (95% CI)** |  |
| **Neurobehavioral development - Autonomic system** | | | | | | | | | | | |
| 3 | randomised trials | serious ^a^ | serious ^b^ | not serious | serious ^c^ | none | 117 | 112 | - | MD **0.83 lower** (1.28 lower to 0.37 lower) | ⨁◯◯◯ VERY LOW |
| **Neurobehavioral development - Motor system** | | | | | | | | | | | |
| 3 | randomised trials | serious ^a^ | serious ^b^ | not serious | serious ^c^ | none | 117 | 112 | - | MD **1.04 lower** (1.58 lower to 0.5 lower) | ⨁◯◯◯ VERY LOW |
| **Neurobehavioral development - State system** | | | | | | | | | | | |
| 3 | randomised trials | serious ^a^ | not serious ^d^ | not serious | serious ^c^ | none | 117 | 112 | - | MD **0.74 lower** (1.06 lower to 0.42 lower) | ⨁⨁◯◯ LOW |
| **Neurobehavioral development - Interaction-attentional system** | | | | | | | | | | | |
| 3 | randomised trials | serious ^a^ | not serious ^d^ | not serious | serious ^c^ | none | 117 | 112 | - | MD **0.48 lower** (0.85 lower to 0.11 lower) | ⨁⨁◯◯ LOW |
| **Neurobehavioral development - Self-regulatory system** | | | | | | | | | | | |
| 3 | randomised trials | serious ^a^ | not serious ^d^ | not serious | serious ^c^ | none | 117 | 112 | - | MD **0.84 lower** (1.17 lower to 0.51 lower) | ⨁⨁◯◯ LOW |
| **Neurobehavioral development - Examiner facilitation** | | | | | | | | | | | |
| 3 | randomised trials | serious ^a^ | not serious ^d^ | not serious | serious ^c^ | none | 117 | 112 | - | MD **1.02 lower** (1.44 lower to 0.6 lower) | ⨁⨁◯◯ LOW |
| **Neurological development** | | | | | | | | | | | |
| 2 | randomised trials | serious ^a^ | serious ^b^ | not serious | very serious ^i^ | none | 72 | 65 | - | MD **-15.0 lower** (-25.28 lower to -4.73 lower) | ⨁◯◯◯ VERY LOW |

#### Explanations

a. Quality of evidence was downgraded by one level as the majority of risk of bias judgements was rated as "unclear" or "high".

b. Quality of evidence was downgraded by one level for inconsistency due to moderate or substantial heterogeneity among studies (40% to 75%).

c. Quality of evidence was downgraded by one level for imprecision as the total number of participants is less than the threshold for continuous outcomes (<400).

d. Quality of evidence was not downgraded for inconsistency as heterogeneity might not be important (<40%).

e. Quality of evidence was downgraded by two levels as the total number of participants is less than the threshold for imprecision (<150).

Developmental Care - Alternative Positioning compared to Standard care

| **Certainty assessment** | | | | | | | **Impact** | **Certainty** |
| --- | --- | --- | --- | --- | --- | --- | --- | --- |
| **№ of studies** | **Study design** | **Risk of bias** | **Inconsistency** | **Indirectness** | **Imprecision** | **Other considerations** |  |  |
| **Neurobehavioral Development** | | | | | | | | |
| 1 | randomised trials | not serious ^a^ | not serious ^b^ | not serious | very serious ^c^ | none | Only one significant effect for the asymmetry subscale (MD 0.88; 95% CI 0.45 1.31; p<0.0001) where infants in treatment group showed less asymmetry than those of the control group. No significant effect was found for the other subscales (attention, handling, quality of movement, regulation, nonoptimal reflexes, stress abstinence, arousal, hypotonicity, hypertonicity, excitability, lethargy. | ⨁⨁◯◯ LOW |

#### Explanations

a. Quality of evidence was not downgraded as the majority of risk of bias judgements was rated as "low".

b. Quality of evidence was not downgraded for inconsistency as there was only one study included in this analysis.

c. Quality of evidence was downgraded by two levels as the total number of participants is less than the threshold for imprecision (<150).

Developmental Care - Positioning and Incubators Covers compared to Standard Care

| **Certainty assessment** | | | | | | | **№ of patients** | | **Effect** | | **Certainty** |
| --- | --- | --- | --- | --- | --- | --- | --- | --- | --- | --- | --- |
| **№ of studies** | **Study design** | **Risk of bias** | **Inconsistency** | **Indirectness** | **Imprecision** | **Other considerations** | **Developmental care (positioning + incubators covers)** | **Standard care** | **Relative (95% CI)** | **Absolute (95% CI)** |  |
| **Neurological development** | | | | | | | | | | | |
| 1 | randomised trials | not serious ^a^ | not serious ^b^ | not serious | very serious ^c^ | none | 76 | 72 | - | RR **0.93 higher** (0.70 higher to 1.22 higher) | ⨁⨁◯◯ LOW |

**Explanations**

a. Quality of evidence was not downgraded as the majority of risk of bias judgements was rated as "low".

b. Quality of evidence was not downgraded for inconsistency as there was only one study included in this analysis.

c. Quality of evidence was downgraded by two levels as the total number of participants is less than the threshold for imprecision (<150).

Parental Participation Programs compared to Standard Care

| **Certainty assessment** | | | | | | | **№ of patients** | | **Effect** | | **Certainty** |
| --- | --- | --- | --- | --- | --- | --- | --- | --- | --- | --- | --- |
| **№ of studies** | **Study design** | **Risk of bias** | **Inconsistency** | **Indirectness** | **Imprecision** | **Other considerations** | **Parental Participation Program** | **Standard care** | **Relative (95% CI)** | **Absolute (95% CI)** |  |
| **Neurobehavioral development** | | | | | | | | | | | |
| 2 | randomised trials | serious ^a^ | very serious ^b^ | not serious | serious ^c^ | none | 145 | 149 | - | MD **5.39 higher** -3.43 lower to 14.20 higher) | ⨁◯◯◯ VERY LOW |

**Explanations**

a. Quality of evidence was downgraded by one level as the majority of risk of bias judgements was rated as "unclear" or "high".

b. Quality of evidence was downgraded by two levels for inconsistency due to considerable heterogeneity among studies (75% to 100%).

c. Quality of evidence was downgraded by one level for imprecision as the total number of participants is less than the threshold for continuous outcomes (<400).

Sensory Stimulation – Tactile stimulation compared to Standard Care

| **Certainty assessment** | | | | | | | **Impact** | **Certainty** |
| --- | --- | --- | --- | --- | --- | --- | --- | --- |
| **№ of studies** | **Study design** | **Risk of bias** | **Inconsistency** | **Indirectness** | **Imprecision** | **Other considerations** |  |  |
| **Neurobehavioral Development** | | | | | | | | |
| 1 | randomised trials | not serious ^a^ | not serious ^b^ | not serious | very serious ^c^ | none | No significant difference between groups for any of the 12 subscales (attention, handling, quality of movement, regulation, nonoptimal reflexes, asymmetric reflexes, stress abstinence, arousal, hypotonicity, hypertonicity, excitability, lethargy). | ⨁⨁◯◯ LOW |

#### Explanations

1. Quality of evidence was not downgraded as the majority of risk of bias judgements was rated as "low".
2. Quality of evidence was not downgraded for inconsistency as there was only one study included in this analysis.

c. Quality of evidence was downgraded by two levels as the total number of participants is less than the threshold for imprecision (<150).

Sensory Stimulation – Multisensory intervention compared to Standard Care

| **Certainty assessment** | | | | | | | **№ of patients** | | **Effect** | | **Certainty** |
| --- | --- | --- | --- | --- | --- | --- | --- | --- | --- | --- | --- |
| **№ of studies** | **Study design** | **Risk of bias** | **Inconsistency** | **Indirectness** | **Imprecision** | **Other considerations** | **Sensorial stimulation (Multisensorial interventions)** | **Standard care** | **Relative (95% CI)** | **Absolute (95% CI)** |  |
| **Neuromotor Development** | | | | | | | | | | | |
| 1 | randomised trials | serious ^a^ | not serious ^b^ | not serious | very serious ^c^ | none | 25 | 25 | - | MD 3**.08 higher** (1.33 higher to 4.83 higher) | ⨁◯◯◯ VERY LOW |
| **Neuromuscular Development** | | | | | | | | | | | |
| 1 | randomised trials | not serious ^d^ | not serious ^b^ | not serious | very serious ^c^ | none | 40 | 40 | - | MD **5.60 higher** (4.65 higher to 6.55 higher) | ⨁⨁◯◯ LOW |

**Explanations**

a. Quality of evidence was downgraded by one level as the majority of risk of bias judgements was rated as "unclear" or "high".

b. Quality of evidence was not downgraded for inconsistency as there was only one study included in this analysis.

c. Quality of evidence was downgraded by two levels as the total number of participants is less than the threshold for imprecision (<150).

d. Quality of evidence was not downgraded as the majority of risk of bias judgements was rated as "low".

Music compared to Developmental Care

| **Certainty assessment** | | | | | | | **№ of patients** | | **Effect** | | **Certainty** | **Importance** |
| --- | --- | --- | --- | --- | --- | --- | --- | --- | --- | --- | --- | --- |
| **№ of studies** | **Study design** | **Risk of bias** | **Inconsistency** | **Indirectness** | **Imprecision** | **Other considerations** | **Music** | **Developmental Care** | **Relative (95% CI)** | **Absolute (95% CI)** |  |  |
| **Neuromotor Development (TIMP)** | | | | | | | | | | | | |
| 1 | randomised trials | serious ^a^ | not serious ^b^ | not serious | very serious ^c^ | none | 18 | 18 | - | MD **0.39 higher** (0.08 higher to 0.70 higher) | ⨁◯◯◯ VERY LOW |  |
| **Neuromotor Development (INFANIB)** | | | | | | | | | | | | |
| 1 | randomised trials | serious ^a^ | not serious ^b^ | not serious | very serious ^c^ | none | 18 | 18 | - | MD 1.89 **higher** (0.42 higher to 3.36 higher) | ⨁◯◯◯ VERY LOW |  |

**Explanations**

a. Quality of evidence was downgraded by one level as the majority of risk of bias judgements was rated as "unclear" or "high".

b. Quality of evidence was not downgraded for inconsistency as there was only one study included in this analysis.

c. Quality of evidence was downgraded by two levels as the total number of participants is less than the threshold for imprecision (<150).

Physical Therapy, Hydrotherapy, Physical Therapy combined to Hydrotherapy compared to Containment

| **Certainty assessment** | | | | | | | **Impact** | **Certainty** |
| --- | --- | --- | --- | --- | --- | --- | --- | --- |
| **№ of studies** | **Study design** | **Risk of bias** | **Inconsistency** | **Indirectness** | **Imprecision** | **Other considerations** |  |  |
| **Neuromotor development** | | | | | | | | |
| 1 | randomised trials | not serious ^a^ | not serious ^b^ | not serious | very serious ^c^ | none | For all interventions, the ANOVA effects were not significant for physical therapy (mean: 50.21) vs. containment (mean: 51.57); hydrotherapy (mean 48.05) vs. containment (mean 51.57); physical therapy combined to hydrotherapy (mean: 52.00) vs. containment (mean: 51.57): p=0.11. | ⨁⨁◯◯ LOW |

**Neuromuscular development**

| 1 | randomised trials | not serious ^a^ | not serious ^b^ | not serious | very serious ^c^ | none | For the neuromuscular development, no significant findings were found for the items of the New Ballard score (p>0.05) and ankle dorsiflexion except for the leg recoil (Dubowitz) which was significantly better for the physical therapy and hyderotherapy groups (p=0.04). | ⨁⨁◯◯ LOW |
| --- | --- | --- | --- | --- | --- | --- | --- | --- |

**Explanations**

a. Quality of evidence was not downgraded as the majority of risk of bias judgements was rated as "low".

b. Quality of evidence was not downgraded for inconsistency as there was only one study included in this analysis.

c. Quality of evidence was downgraded by two levels as the total number of participants is less than the threshold for imprecision (<150).
